# Efficient Preparation of Biodiesel Using Sulfonated *Camellia oleifera* Shell Biochar as a Catalyst

**DOI:** 10.3390/molecules29122752

**Published:** 2024-06-09

**Authors:** Zhimin Yang, Yu Wang, Xichang Wu, Wenxuan Quan, Qi Chen, Anping Wang

**Affiliations:** 1 Key Laboratory for Information System of Mountainous Area and Protection of Ecological Environment of Guizhou Province, Guizhou Normal University, Guiyang 550025, China; yangzhimin@gznu.edu.cn (Z.Y.); wangyu@gznu.edu.cn (Y.W.); wuxichang@gznu.edu.cn (X.W.); 2 School of Materials and Architectural Engineering, Guizhou Normal University, Guiyang 550025, China; qichen@gznu.edu.cn

**Keywords:** biochar, biodiesel, sulfonation, *Camellia oleifera* fruit shell, heterogeneous catalyst

## Abstract

This study prepared sulfonated *Camellia oleifera* shell biochar using *Camellia oleifera* shell agricultural waste as a carbon source, and evaluated its performance as a catalyst for preparing biodiesel. The biochar obtained from carbonizing *Camellia oleifera* shells at 500 °C for 2 h serves as the carbon skeleton, and then the biochar is sulfonated with chlorosulfonic acid. The sulfonic acid groups are mainly grafted onto the surface of *Camellia oleifera* shell biochar through covalent bonding to obtain sulfonic acid type biochar catalysts. The catalysts were characterized by Scanning Electron Microscope (SEM), X-ray diffraction (XRD), Nitrogen adsorption-desorption Brunel-Emmett-Taylor Theory (BET), and Fourier-transform infrared spectroscopy (FT-IR). The acid density of the sulfonated *Camellia oleifera* fruit shell biochar catalyst is 2.86 mmol/g, and the specific surface area is 2.67 m^2^/g, indicating high catalytic activity. The optimal reaction conditions are 4 wt% catalyst with a 6:1 alcohol to oil ratio. After esterification at 70 °C for 2 h, the yield of biodiesel was 91.4%. Under the optimal reaction conditions, after four repeated uses of the catalyst, the yield of biodiesel still reached 90%. Therefore, sulfonated *Camellia oleifera* shell biochar is a low-cost, green, non-homogeneous catalyst with great potential for biodiesel production by esterification reaction in future development.

## 1. Introduction

As global industrialization accelerates, the demand for fossil fuels continues to rise. However, fossil fuels, once consumed, cannot be formed within a short period of time and are therefore categorized as non-renewable resources. At present, although there are still some global reserves of fossil fuels, mankind may face energy depletion in the future as fossil fuels continue to be consumed. In addition to the problem of resource depletion, the burning of fossil fuels poses serious environmental hazards. Fossil fuels produce carbon dioxide during their use, enhancing the greenhouse effect and contributing to global warming. Global carbon dioxide emissions reached 30.6 billion tons in 2020 as a result of fossil fuels being the main source of energy. Carbon dioxide emissions are expected to increase by 50 per cent in 2030 [1]. In addition, the combustion process releases pollutants such as sulfides, nitrogen oxides, and particulate matter, which not only pose a threat to human health, but also lead to environmental problems such as acid rain, urban smog, and the degradation of air quality [2,3,4,5]. In order to solve the energy crisis and environmental problems, researchers are committed to developing new energy sources that can replace fossil fuels, and biodiesel is considered a new energy source that can replace fossil diesel due to its wide source of raw materials, low sulfur content, low greenhouse gas emissions during combustion, and renewable advantages [6,7,8,9].

Biodiesel is a class of advanced esters of long-chain fatty acids, produced by the catalytic esterification or transesterification of animal fats or vegetable oils with alcohols [10,11]. Catalysts for biodiesel production are mainly divided into acid catalysts, base accelerators, and enzyme catalysts [12]. However, the use of enzymes as biodiesel catalysts makes it difficult for enzymes to be used as catalysts in the industrial production of biodiesel due to their high cost and poor stability and reusability [13]. In more recent years, researchers have been constantly looking for cheap, non-edible crude oil as a biodiesel feedstock, for use in cooking oil, jatropha oil, castor oil, etc. However, the acid value of these oils is high, and the direct use of alkali catalysts without treatment will produce a saponification process, which will lead to a reduction in the yield of biodiesel so that acid catalysts have a wider application in the process of biodiesel production [14,15,16,17,18]. Acid catalysts are unaffected by free fatty acids and can catalyze transesterification and transesterification reactions to form biodiesel [19]. Homogeneous acid catalysts not only cause corrosion to the reaction apparatus during use but also pollute the environment [20]. Currently, non-homogeneous acid catalysts have attracted a wide range of scholars to study them because of their advantages, such as being environmentally friendly, easy to separate, and reusable [21].

Some researchers have proposed sulfonated catalysts, whereby biochar is prepared from agricultural waste, and then sulfonated biochar is functionalized to produce sulfonated carbon-based catalysts as low-cost renewable “green catalysts” [22]. Sulfonation functionalization refers to the grafting of sulfonic acid groups onto biochar, which greatly improves the catalytic effect due to the incorporation of sulfonic acid groups (—SO_3_H), as well as pre-existing phenolic (—OH) and carboxylic (—COOH) groups in sulfonated charcoal-based catalysts [23]. Sulfonated biochar is one of a number of solid acid catalysts, with excellent stability, efficient catalytic performance, and a low price; sulfonated biochar is a solid catalyst that can replace the liquid sulfuric acid catalysts [24]. Agricultural waste biomass is generally treated by burning or natural decay, and the use of agricultural biomass charcoal as a carbon base can solve the problem of its treatment, as well as obtaining a cheap catalyst feedstock [25]. Agricultural waste biomass that have been studied in the past include coconut shells, bagasse, corn cobs, and palm kernel shells, which share a common characteristic of high lignin and cellulose content [26]. *Camellia oleifera* is a natural oil that grows in the southern region of China, mainly in Hunan, Jiangxi, Zhejiang, and Hainan [27]. *Camellia oleifera* fruit shell (COS) is the waste produced during the processing of COS into tea oil. China produces a large amount of fruit shell waste every year, and the fruit shells contain a large amount of lignin and cellulose, which can be used to make biochar [28]. 

In this study, *Camellia oleifera* fruit shell, which includes a high content of lignin and cellulose, has been selected as the charcoal source for sulfonated biochar. The *Camellia oleifera* fruit shell biochar (COSC) has good adsorption properties, and the COSC is a multilayered structure. These structures can provide more active sites after sulfonation functionalization. Sulfonation of COSC using chlorosulfonic acid was used to make a sulfonated *Camellia oleifera* fruit shell biochar (COSC-SO_3_H) catalyst. The esterification reaction of oleic acid with methanol was chosen as a model to evaluate its catalytic performance. A series of characterizations were performed to determine the structure and performance of the catalyst.

## 2. Results and Discussion

### 2.1. FT-IR and XRD Analysis

**Figure 1:**
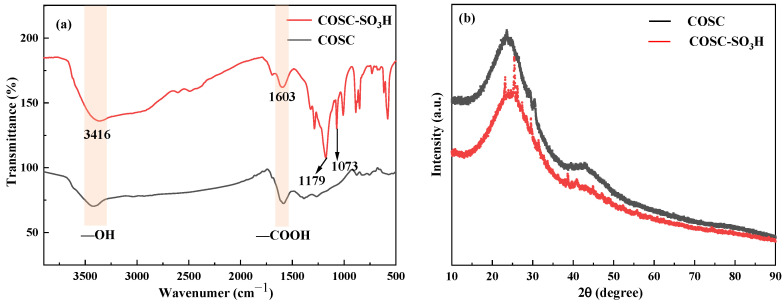
(**a**) FT-IR spectra of COSC-SO_3_H, COSC, and (**b**) X-ray diffractogram.

The infrared spectra of COSC and COSC-SO_3_H are shown in Figure 1a. The results showed that the sulfonation process successfully introduced sulfonic acid groups on the COSC. From the figure, it can be seen that the *Camellia oleifera* biochar showed similar IR absorption bands at 3416 cm^−1^ and 16,036 cm^−1^ before and after sulfonation of *Camellia oleifera* shell biochar. These peaks correspond to the stretching vibrations of hydroxyl (—OH) and carboxyl (—COOH) groups, which suggests the formation of these oxygen-containing functional groups during carbonization [29]. The sulfonated oleaginous shell biochar showed a new absorption band at 1179 cm^−1^, which was attributed to the asymmetric stretching vibration of the—SO_3_H group. In addition, a symmetric stretching vibration absorption band of the—SO_3_H group was detected at 1073 cm^−1^ [30]. 

As shown in Figure 1b, the XRD patterns before and after sulfonation of COSC are shown. There is no significant difference between the XRD plots of both COSC and COSC-SO_3_H, and the distribution trends are approximately the same. This indicates that sulfonation has little effect on the microstructure of the carbon-based materials. In the figure, it can be seen that both of them show a strong and broad C (002) diffraction peak at 2θ of 15°–30°, which is the characteristic diffraction peak of the amorphous carbon structure, and the amorphous structure here is due to the irregular stacking of parallel layers [31]. The wider the diffraction peak width is, the greater the degree of disorder, indicating that the disorder of the carbon structure is weakened after sulfonation [32]. In addition, there is a broad and weak C (101) derivation diffraction peak at 2θ at 40°–50° due to the α-axis in the graphite structure and the low carbon structure graphitization, which ensures the nucleophilic surface properties of the loaded sulfonic acid groups [33]. The diffraction peak heights and widths in the figure show that the internal structures of the COSC and COSC-SO_3_H biochar are disordered and amorphous.

### 2.2. Pore Structure Analysis

**Figure 2:**
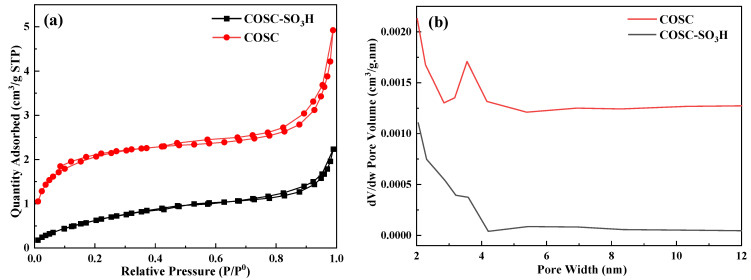
(**a**) Nitrogen adsorption-desorption, (**b**) pore size distribution.

As shown in Figure 2a, both the adsorption isotherms before and after the sulfonation treatment of COSC belong to the type II isotherm, which is a reversible process of monolayer adsorption that usually occurs on non-porous or macroporous solids. The inflection point on the isotherm marks the transition from monolayer to multilayer adsorption, which implies that the monolayer adsorption is saturated and multilayer adsorption is subsequently initiated. From Figure 2b, it can be seen that the pore size of the COSC was mainly distributed in 2.8–5.3 nm, while the pore size of the COSC-SO_3_H was mainly distributed in 3.2–4.2 nm. As shown in Table 1, it can be deduced from the isotherms of the two, as well as the specific surface area and pore volume, that the pore size of the two is mainly dominated by the mesoporous structure accompanied by a part of the macroporous structure. Therefore, both untreated and COSC-SO_3_H showed strong adsorption capacity.

### 2.3. SEM Analysis

**Figure 3:**
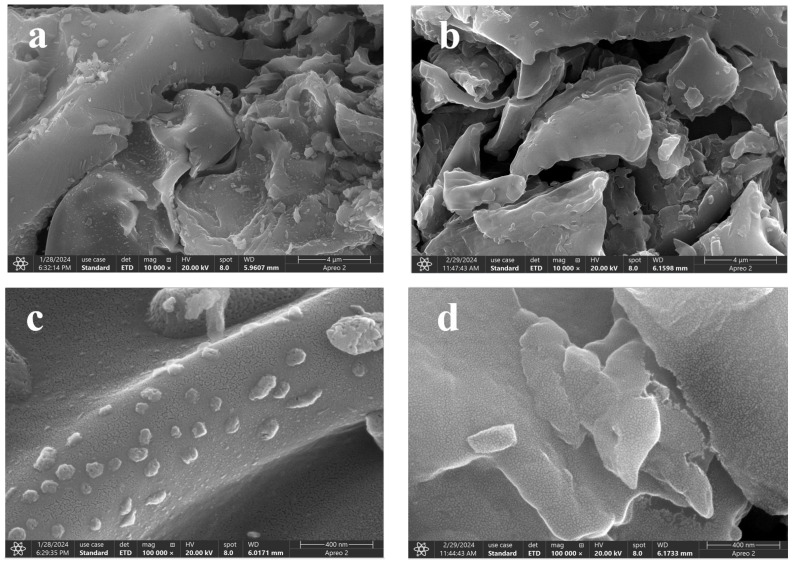
SEM images of COSC (**a**,**c**), and COSC-SO_3_H (**b**,**d**).

The surface characteristics of COSC and COSC-SO_3_H were observed by SEM analysis, as shown in Figure 3. From Figure 3a,c it can be seen that the surface of the COSC was uneven, and the crevices were generated due to the lignocellulose in the COS after high-temperature pyrolysis, which made the COSC have a large specific surface area. From Figure 3b,d it can be seen that the surface of COSC after sulfonation is smooth and flat, and the crevices increase. It shows an amorphous shape before and after sulfonation, which agrees with the results of the XRD analysis.

### 2.4. Biodiesel Yield Analysis

**Figure 4:**
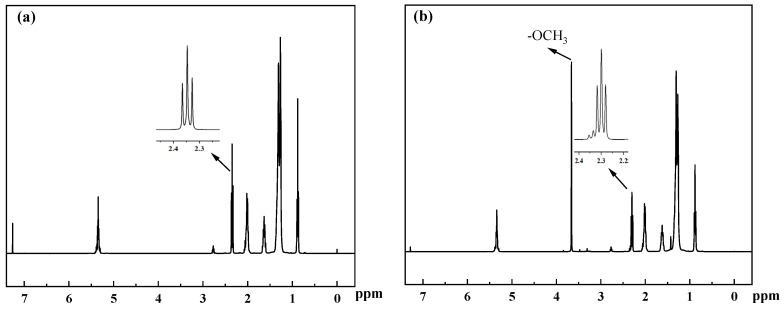
(**a**) ^1^H spectrum of oleic acid, (**b**) ^1^H spectrum of methyl oleate.

NMR is an effective tool for the detection of biodiesel. In the detection process, deuterated chloroform was used as the solvent, and Tetramethylsilane (TMS) as the internal standard. The yield from methyl oleate is determined through the ratio of the peak area of the substrate before and after the reaction to the peak area of the product. As seen in Figure 4a, the hydrogen spectrum of oleic acid shows a very perfect α—CH_2_ proton peak at around 2.35 ppm. The hydrogen spectra of methyl oleate are shown in Figure 4b. The hydrogen spectra of methyl oleate and oleic acid are almost the same, and there is only a new peak at 3.66 ppm for methyl oleate, which is the hydrogen peak of —OCH_3_ produced by methanol through esterification. When all oleic acid is converted to methyl oleate, the —OCH_3_ peak area is 1.5 times the area of the α—CH_2_ peak. Therefore, the ratio of —OCH_3_ to α—CH_2_ peak area was chosen to calculate the yield. The peak area of α—CH_2_ at 2.3 ppm was calibrated to 1. The ratio of the peak area of —OCH_3_ at 3.66 ppm to that of α—CH_2_ at 2.3 ppm was used to calculate the yield of methyl oleate. The yield is calculated from Equation (1):

(1) $$C=(2A_{Me}/3A_{CH_{2}})$$

where *A_Me_* denotes the area of integration of methoxyhydrogen at 3.66 ppm and $A_{CH_{2}}$ denotes the area of integration of α—CH_2_ at 2.30 ppm.

### 2.5. Optimization of the Esterification Reaction Process 

**Figure 5:**
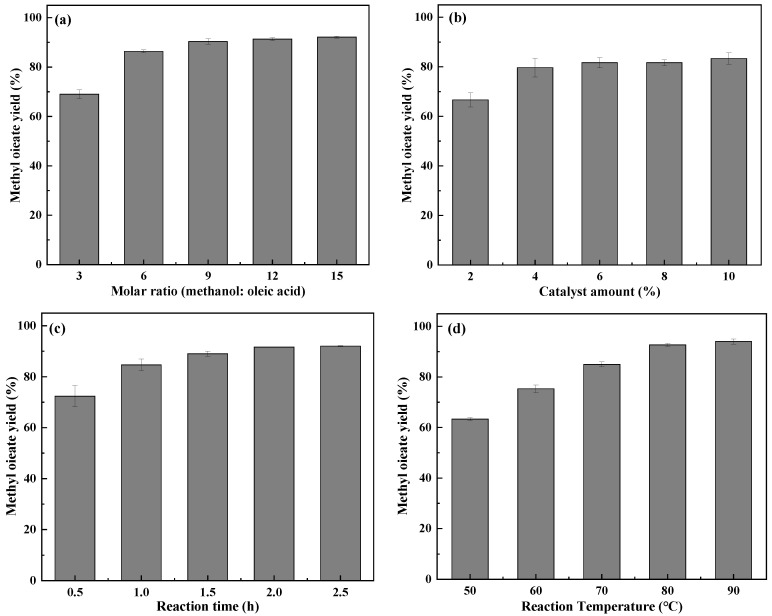
(**a**) Optimization of the molar ratio of methanol/oleic acid, (**b**) catalyst dosage, (**c**) reaction time, (**d**) reaction temperature.

The effect of the molar ratio between methanol and oleic acid (reaction conditions: temperature is 70 °C, reaction time is 1 h, and catalyst content is 6 wt%) on the conversion rate of oleic acid in the esterification reaction is shown in Figure 5a. The conversion of oleic acid showed an increasing and then leveling off trend with the increasing amount of methanol in the reaction. The yield of biodiesel was 86.3% when the molar ratio was 6:1. Oleic acid esterification with methanol is a reversible reaction, and the esterification reaction is limited when there is insufficient methanol in the reaction system. When the molar ratio is 3, the conversion is 69.0%. When the molar ratio reaches 6 and above, the conversion remains essentially the same, which is due to the excess methanol reducing the effective collision of reactant molecules in the reaction system [33]. The optimum molar ratio for its chemical reaction is 6:1.

The effect of catalyst content (reaction conditions: methanol/oleic acid of 6:1, temperature of 60 °C, reaction time of 1 h) on biodiesel yield is shown in Figure 5b. With the increasing catalyst content, the oleic acid conversion rate showed a trend of increasing and then leveling off. The catalyst content reached 4 wt%, at which point the oleic acid conversion was 79.7%, and this point indicated the optimum catalyst content. When the catalyst content increases, its conversion rate will also increase, but the continuous increase in catalyst content will limit the mass transfer of the reaction system, so the conversion rate remains unchanged when the catalyst content continues to increase [17].

The influence of reaction time (reaction conditions: methanol/oleic acid is 6:1, temperature is 60 °C, catalyst content is 4 wt%) on the transformation rate of oleic acid is shown in Figure 5c. The esterification reaction requires sufficient time to allow contact between the liquid reactants and the solid catalyst to ensure that the esterification reaction on the surface of the catalyst proceeds thoroughly. As the reaction time increases, the biodiesel yield increases, and finally the yield levels off. When the reaction time reaches 2 h, the increase in its conversion rate is slow and the conversion rate of the reaction at 2 h is 84.7%.

The Impact of reaction temperature on (reaction conditions: alcohol/oleic acid is 6:1, reaction time is 1 h, catalyst content is 4 wt%) the conversion of oleic acid is shown in Figure 5d. The esterification efficiency increased continuously with the increase in temperature. At reaction temperatures above 80 °C, the reaction conversion rises slowly. This may be due to the vaporization of methanol at too high a reaction temperature and the decrease in the concentration of methanol in the reaction system, which counteracts the increase in efficiency [34]. Thus, the trend of increasing efficiency becomes slow. The optimum temperature of the reaction was reached at 70 °C with a conversion of 85.0%.

Through the analysis of the above data, the optimal reaction conditions for the polyphase solid acid-catalyzed esterification reaction of oleic acid with methanol were optimized: the catalyst dosage was 4 wt%, the molar ratio of methanol to oleic acid was 6:1, the esterification reaction time was 2 h, and the esterification reaction temperature was 70 °C, and the esterification efficiency reached 91.4%. Three replicate experiments were carried out under the optimized conditions, and the esterification efficiencies were 91.6%, 91.8%, and 91.1%, respectively.

### 2.6. Water Tolerance and Thermal Filtration Analysis 

**Figure 6:**
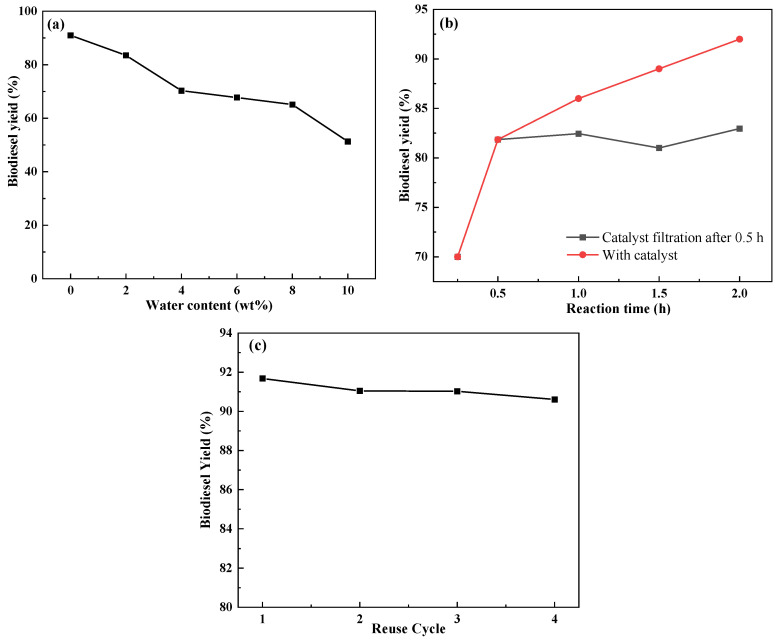
(**a**) Water tolerance experiment for the esterification reaction of oleic acid at 70 °C, (**b**) thermal filtration experiment for the esterification reaction of oleic acid at 70 °C, (**c**) reusability of COSC-SO_3_H catalysts for biodiesel production at 70 °C.

Water in the esterification reaction is a by-product of this reaction, which will deactivate the active sites of acidic catalysts with poor water tolerance, ultimately reducing the efficiency of the reaction. Therefore, the study of water tolerance of catalysts is particularly important. The results of the water tolerance experiments of the catalysts are shown in Figure 6a. It can be seen that the catalyst yields 83.5% of methyl oleate at 2 wt% water content in the reaction system, and when the water content in the reaction system is at 4 wt%, the yield of methyl oleate decreases to 70.3%. The experimental results showed an excellent catalytic performance of the modified catalyst at low water content of the reaction system. Figure 6b shows the results of thermal filtration experiments of the catalyst. After 0.5 h of reaction of the catalyst-containing system, the catalyst was filtered and removed, and then the reaction was continued three different times, and finally the yields of methyl oleate with different reaction times were measured. From Figure 6b, it can be seen that the yield of methyl oleate did not increase when the catalyst was removed, which proves the non-homogeneous nature of the catalyst. The biodiesel yield of COSC-SO_3_H remained at 90% in four consecutive cycles, as shown in Figure 6c. This indicates that the catalyst has good reusability.

### 2.7. Reaction Kinetics Studies

The catalytic esterification of oleic acid with methanol using COSC-SO_3_H can be expressed as follows:

$${\mathrm{CH}}_{3}({\mathrm{CH}}_{2}{)}_{7}\mathrm{CH}=\mathrm{CH}({\mathrm{CH}}_{2}{)}_{7}\mathrm{COOH}+{\mathrm{CH}}_{3}\mathrm{OH}\;\overset{Catalyst}{\Leftrightarrow }{\mathrm{CH}}_{3}({\mathrm{CH}}_{2}{)}_{7}\mathrm{CH}=\mathrm{CH}({\mathrm{CH}}_{2}{)}_{7}{\mathrm{COOCH}}_{3}+H_{2}O$$

The molar ratio of methanol to oleic acid at the best reaction conditions selected through the optimization of the esterification reaction conditions was 6:1, and the amount of methanol in the reaction system was much larger than that of oleic acid, so that the change in the concentration of methanol could be ignored during the reaction. The mass transfer resistance during this reaction is negligible and can be viewed as pseudo-primary kinetics. The simplified kinetic equation of the primary reaction is shown in Equation (2):

(2) $$r=\frac{-dC_{A}}{dt}=kC_{A}$$

where *r* is the reaction rate constant, *C_A_* is the oleic acid concentration, and *k* is the rate constant.

Taking the natural logarithm of *C_A_*, Equation (3) is obtained:

(3) $$-In\left(1-X\right)=kt$$

where *X* is the biodiesel yield, *t* is the reaction time, and *k* is the reaction rate constant.

Calculating the activation energy from Arrhenius’ equation:

(4) $$lnk=-\frac{E_{a}}{RT}+lnA$$

where *A* is the antecedent factor, *E_a_* is the activation energy, and *T* is the reaction temperature.

**Figure 7:**
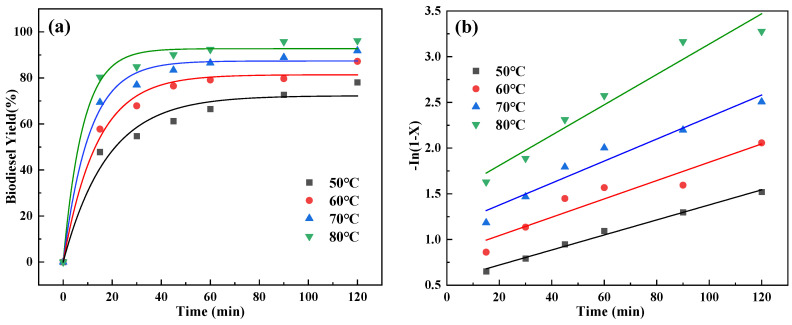

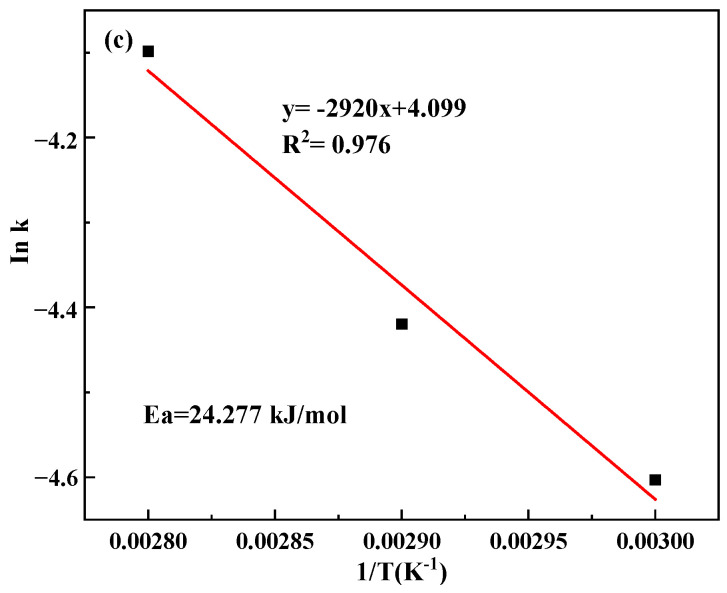
(**a**) Reaction process at different temperatures, (**b**) linear plots of reaction time and −Ln(1 − *X*) at different reaction temperatures, (**c**) LnK plotted against 1/T.

From Figure 7a, it can be seen that the yield of the esterification reaction varies with time at different temperatures and the general trend is that the increase in its biodiesel yield subsequently tends to plateau with the increase in time. From Figure 7b, it can be seen that the rate constant of the reactions increases with an increasing temperature and has a nice linear relationship, so the biodiesel yield also follows the pseudo-primary reaction kinetics [32]. The activation energy *E_a_* for esterification reaction can be calculated as 24.28 kJ/mol by Arrhenius’ equation. The activation energy of this catalyst is lower than that of previously studied catalysts, as shown in Figure 3. Due to the low activation energy of the reaction, it can be deduced that sulfonation of oleaginous tea husk biochar reduces the activation energy required for the reaction. From Figure 7c, it can be seen that the COSC-SO_3_H catalyst has high activity (Table 2 and Table 3).

## 3. Experimental Materials and Method

### 3.1. Materials

*Camellia oleifera* fruit shells were sourced from Tianzhu County, Guizhou Province, China. Anhydrous ethanol (AR, 99.7%) and oleic acid (AR, 98%) were purchased from Beijing InnoChem Science & Technology Co., Ltd. (Beijing, China). Sodium hydroxide (AR, 97%) and dichloromethane (AR, 99%) were purchased from Shanghai Macklin Biochemical Technology Co., Ltd. (Shanghai, China). Potassium hydroxide (AR, 85%) was purchased from Shanghai Aladdin Biochemical Technology Co., Ltd. (Shanghai, China). Petroleum ether (AR, 98.5%) and anhydrous methanol (AR, 99.5%) were purchased from Tianjin Zhiyuan Chemical Reagent Co., Ltd. (Tianjin, China). Chlorosulfonic acid (AR, 99%) was purchased from Jiuding Chemical (Shanghai) Technology Co., Ltd. (Shanghai, China). All reagents were unpurified and used directly.

### 3.2. Sulfonation of Biochar from Camellia oleifera Fruit Shell

The dried COS was pulverized with a pulverizer until it could pass through a 40-mesh sieve, and the pulverized COS was placed in a tube furnace, adjusted to a temperature of 500 °C, and heated at this temperature for 2 h to obtain COSC powder. Add 5.0 g of biochar powder of COS in a round-bottomed flask with 70 mL of dichloromethane under magnetic stirring to make the charcoal powder evenly dispersed in the dichloromethane, add 10 mL of dichloromethane and 0.75 mL of chlorosulphonic acid in a constant-pressure funnel, slowly dripping into the round-bottomed flask and stirring at room temperature; continue stirring at 25 °C for 1.5 h after the constant pressure funnel has finished dripping. The sulfonated *Camellia oleifera* fruit shell charcoal powder was washed with dichloromethane until neutral. Finally, the washed charcoal powder was put into an oven set at 45 °C and dried for 12 h in order to obtain a COSC-SO_3_H catalyst.

### 3.3. Determination of the Catalyst Acid Density

Acid density determination of catalysts was performed using the neutralized acid-base titration method [36]. Forty mg of catalyst was added to 20 mL of NaCl (0.1 M) solution and the reaction was magnetically stirred at 25 °C for 24 h; the magnet was removed and centrifuged to separate the solids from the solution, the solution was placed in a conical flask with 5 drops of phenolphthalein indicator and titrated with 0.01 mol/L NaOH solution in water, and the volume of NaOH used was recorded.

Calculate the acid density of the catalyst by using the following Equation (5).

(5) Acid density=[c(NaOH)×V(NaOH)]/m(catalyst)

where *V* is the volume of NaOH standard solution mL, *c* is the concentration of NaOH standard solution mol/L, and *m* is the mass of specimen g.

The acid density of COSC-SO_3_H was estimated to be 2.85 mmol/g. 

### 3.4. Esterification Reactions and Analytical Methods

The experimental method was slightly modified from the previous experimental method [37]. Methanol, oleic acid, and catalyst are added to a 15 mL glass pressure-resistant flask and then heated for esterification reaction in a water bath with magnetic stirring under 400 rpm. After reaching a specific reaction time, a catalyst was removed by centrifugation (8000 rpm, 3 min) with extraction with petroleum ether, and unreacted methanol as well as petroleum ether and water were removed by evaporation under reduced pressure. The test was repeated three times at the same reaction conditions, and the molar ratio (methanol/oleic acid) is 3–15, the catalyst dosage is 2–10 wt%, the reaction temperature is 60–100 °C, and the reaction time is 1–5 h, which are optimized during the reaction process. The yield is determined by a Nuclear Magnetic Resonance (NMR) ^1^H spectrum.

### 3.5. Catalyst Characterization

The functional groups of the catalysts was determined using a Nicolet-iS10 infrared spectrometer (Thermo Fisher Scientific, Waltham, MA, USA), and the samples were scanned from 500 cm^−1^ to 4000 cm^−1^ to obtain infrared absorption data. A Thermo Fisher Scientific model Apreo 2 SEM was used to observe the catalyst morphology. The physical properties (specific surfaced area, pore volume, and pore size) of the catalysts were analyzed using a nitrogen adsorption-desorption unit, Micro for TriStar II Plus 3030, from Micro, Greensboro, NC, USA. X-ray diffraction was recorded using a Thermo Fisher Scientific Escalab 250Xi X-ray diffractometer to characterize the crystal morphology of the catalysts, with a scan rate of 2°·min^−1^ and a scanning range of 10° to 90°.

## 4. Conclusions

The experimental results showed that the conversion rate to oleic acid could reach 91% at specific reaction conditions, i.e., catalyst dosage of 4 wt%, alcohol-oil molar ratio is 6:1, temperature is 70 °C, and the reaction time is 2 h. Moreover, the catalyst still achieved a biodiesel yield of 91.4% after being reused four times under optimal reaction conditions. This result indicates that the COSC-SO_3_H catalyst has high catalytic efficiency. In addition, the structural characterization of the catalyst reveals its high activity, high acid density, multiple gaps, and porosity, which are advantageous for catalyzing the reaction. High acid densities provide for more active sites, while multiple crevices and porosity help to increase the specific surface area of the catalyst, thereby increasing reaction rates. As a waste product of widely grown crops, COS is less expensive. Therefore, the development of COSC-SO_3_H catalyst is not only environmentally friendly but also economically attractive as a promising biodiesel catalyst. In summary, the study of oil-tea-shell-based solid acid catalysts offers a new possibility for biodiesel production, and its high efficiency and low cost make it an important commercial option in the field of sustainable energy.

## Figures and Tables

**Table 1:** COSC and COSC-SO_3_H N_2_ adsorption-desorption data.

Sample	BET Surface Area (m^2^/g)	Pore Volume (mm^3^/g)	Pore Size (nm)
COSC	7.6	5.2	10.5
COSC-SO_3_H	2.7	3.4	6.0

**Table 2:** COSC-SO_3_H reaction rate constant in the reaction.

Temperature (°C)	Reaction Rate Constant, k′ (1/min)	Coefficient of Determination (R^2^)
50	0.0166	0.951
60	0.0121	0.949
70	0.0100	0.907
80	0.00822	0.993

**Table 3:** Comparison of activation energy.

Catalysts	Activation Energy	Reference
*Cocos nucifera* (coconut) husk (ACH-SO_3_H)	68.83 kJ/mol	Rhithuparna D et al. [32]
ZP-P[SIH]-2	29.82 kJ/mol	Hu Pan et al. [7]
30%Sn-MMT-SO_3_H	56.98 kJ/mol	Long Chen et al. [35]
COSC-SO_3_H	24.28 kJ/mol	This work

## Data Availability

Data are contained within the article.
